# “Oops…a Beaver Again!” Eurasian Beaver *Castor fiber* Recorded by Citizen-Science in New Areas of Central and Southern Italy

**DOI:** 10.3390/ani13101699

**Published:** 2023-05-20

**Authors:** Giovanni Capobianco, Andrea Viviano, Giuseppe Mazza, Gianmarco Cimorelli, Angelo Casciano, Alessandro Lagrotteria, Romina Fusillo, Manlio Marcelli, Emiliano Mori

**Affiliations:** 1 Associazione per la Ricerca, la Divulgazione e l’Educazione Ambientale (ARDEA), Via Ventilabro 6, 80126 Naples, Italy; gio.cap@hotmail.it; 2 Consiglio Nazionale delle Ricerche, Istituto di Ricerca sugli Ecosistemi Terrestri, Via Madonna del Piano 10, 50019 Florence, Italy; andrea.viviano@unifi.it; 3 CREA Research Centre for Plant Protection and Certification (CREA-DC), Cascine del Riccio, Via Lanciola 12/a, 50125 Florence, Italy; giuseppe.mazza@crea.gov.it; 4 National Biodiversity Future Center, 90133 Palermo, Italy; 5 Associazione Amici del Volturno e dei suoi Affluenti, Via Giuseppe Laurelli 12, 86170 Isernia, Italy; g.cimorelli1997@gmail.com; 6 Free-Lance, Via Piano degli Usci 20, 84020 Salerno, Italy; angelo.casciano11@libero.it; 7 Department of Biological Sciences, Università del Piemonte Orientale, Via G. Ferraris 107, 13100 Vercelli, Italy; lagrotterialessandro@live.it; 8 Lutria sas Wildlife Research and Consulting, Via Stefano Oberto 69, 00173 Roma, Italy; romina.fusillo@lutria.eu (R.F.); manlio.marcelli@lutria.eu (M.M.)

**Keywords:** *Castor fiber*, Central-Southern Italy, citizen science, range expansion, river ecosystems, unauthorised releases

## Abstract

**Simple Summary:**

Eurasian beavers have been released throughout Europe since the 1900s, with most introductions following the international reintroduction guidelines. Conversely, unauthorised releases have occurred in several countries, e.g., in Spain, Belgium, Scotland, and Central Italy (Tuscany and Umbria regions). In March 2023, other unequivocal presence signs were also detected in southern regions (Molise and Campania), confirming the local presence of beavers. Moreover, two more occurrence sites were recorded in Central Italy (Abruzzi). Despite representing a native species, unauthorised releases of beavers raise management problems, emphasizing the importance of tight monitoring to clarify the actual distribution of this large rodent in these areas.

**Abstract:**

The Eurasian beaver *Castor fiber* was once present in the Palearctic, ranging from the western Iberian Peninsula to northwestern China. In the Middle Ages, this rodent underwent a severe decline in population due to habitat loss, hunting for fur and meat, and the demand for castoreum. At the beginning of 1900, the range of the Eurasian beaver was limited to scattered refugia in Eurasia. Since 1920, legal protection, reintroduction events, and natural spread triggered the recovery of the species in most of its original range. In March 2021, the presence of the Eurasian beaver in Central Italy (Tuscany and Umbria regions) was confirmed through camera trapping after the detection of unequivocal signs of presence (i.e., gnawed trunks). Recordings are located about 550 km south of the known range of this species, thus suggesting that the presence of beavers in Tuscany and Umbria might be due to a local unauthorised reintroduction. In this work, we also reported the presence of beavers in the Abruzzi region and in Southern Italy (Molise-Campania regions), over 380 km in a straight line south to the southernmost record of beaver presence in Central Italy.

## 1. Introduction

The Eurasian beaver *Castor fiber* Linnaeus, 1758, once inhabited a significant portion of the Palearctic, ranging from the western Iberian Peninsula to northwestern China, throughout several riparian habitat types in forests, tundra, and steppe [1]. In Medieval times, this rodent’s presence dramatically declined due to hunting for fur and meat, habitat loss, and the demand of “castoreum” [2,3]. At the start of the 20th century, the range of the Eurasian beaver was limited to scattered refugia between France and the Far East, with less than 1200 individuals. Since 1920, legal protection measures have been implemented, which together with reintroduction events and natural spread, have led to the recovery of the species in most parts of its original range, up to a minimum population estimate of about 1.5 million individuals in 2020 [1]. Currently, the Eurasian beaver shows reproductive populations in most of its original range, apart from the southern Balkan Peninsula and, possibly, Portugal [1,4,5].

Salari et al. [6] summarised that *C. fiber* occurred in the Early Pleistocene in most Italian regions, between the Alpine chain and the Volturno River in Molise (Southern Italy). Reintroduction programs that occurred in neighbouring countries (Austria and Switzerland) between the 1970s and the 1990s have triggered a widespread recolonisation [1]. Since 2018, a single Eurasian beaver has been present in Friuli Venezia Giulia (NE Italy), most likely due to natural dispersal from Austria [7]. So far, no authorised beaver reintroduction has ever occurred in Italy. Later, in November 2020, another individual beaver was camera trapped in Alto Adige, near the Austrian border, where the last beaver seen there had been killed in late 1500 [6,8]. In this area, two individual beavers have been observed [8]. In March 2021, two wildlife managers and some members of the provincial police noted some gnawed trunks, i.e., unequivocal signs of beaver presence, in two areas of central Italy, the former in the provinces of Grosseto and Siena (Tuscany) and the latter between the provinces of Arezzo (Tuscany) and of Perugia and Terni (Umbria) [8,9]. Since these sightings are located about 550 km south of the Austrian border, the presence of the beavers in Tuscany and Umbria could be the result of unauthorized reintroductions. Even in the case of legal introductions or reintroductions, the International Union for the Conservation of Nature (IUCN) strongly discourages operations conducted with no feasibility study and no genetic analysis of released stock, because of the damage that released animals may cause to native ecosystems [10]. Accordingly, the recent update of the Italian Red Lists reports the Eurasian beaver as “Not Applicable,” as the only reproductive populations are most likely unofficially released [11]. The activity of beavers is known to change vegetation structure to a great extent, in turn influencing other components of the ecosystems, including the diversity and abundance of invertebrates, amphibians, and wading birds [12,13]. Beaver alteration of heterogeneity and connectivity of habitats (i.e., through the creation of dams and lodges [12,13,14,15,16]) needs further research in our study area [13]. However, beavers have also been deliberately introduced to several countries to increase local biodiversity [14]. Besides causing potential damage to crops, beavers are also reported to increase native biodiversity and shelter sites, to improve the hydrogeological status of European rivers, and to mitigate environmental pollution [12,13,14,15,16]. In Central Italy, the benefits of beaver presence are reported for at least 17 native animal species, including four species of European conservation concern and one Italian endemic small mammal [16]. Furthermore, the Eurasian beaver is listed within the Annexes of the Habitats Directive (92/43/EEC), and, if it is established in Italy, any natural range expansion of this rodent would require the completion of the standard forms provided for each six-year reporting period [17,18]. An updated monitoring of the distribution of the Eurasian beaver is required both for conservation and for management issues.

In 2022–2023, the research project “Rivers with Beavers” was conducted with the aim of monitoring distribution and population size, as well as the potential impacts and genetics of Eurasian beavers in Central Italy. After the first recorded sighting in Central Italy, a widespread media campaign was triggered with over 30 newspaper/tabloid magazine articles, which in turn led to an increase of recorded sightings, which were sent to the research team of “Rivers with Beavers” or the project’s Facebook page. Citizen science initiatives involve common citizens in data collection, always followed by the validation by experts. Projects involving common citizens are increasing worldwide and may store a huge amount of data on species occurrences, thus providing scientists with paramount information to develop strategies for wildlife conservation and management [19,20]. Moreover, research and monitoring of protected species strictly associated with river ecosystems, such as the Eurasian otter *Lutra lutra*, represent another important source of data about the presence of beavers.

In this study, we report the results of four records of beaver sightings obtained through citizen science and a camera trap photo of a beaver, confirming the presence of the Eurasian beaver in Southern Italy, over 350 km from the nearest known sighting previously recorded in Italy.

## 2. Materials and Methods

We collected beaver occurrences through the Facebook page of the project “Rivers with Beavers” (see Acknowledgements) between January 2022 and April 2023. This Facebook page includes photos of signs of presence (gnawed trunks, excrements, and footprints), as well as camera-trapping data showing diagnostic features of Eurasian beavers. The Facebook page (available at: https://www.facebook.com/riverswithbeavers/; accessed on 17 April 2023) has over 1260 followers in Italy, which may have increased citizen awareness on beaver occurrence and behaviour. Therefore, new presence data were collected on the Facebook page through a citizen science approach, based on photos of individual beavers and/or signs of presence. Records confirmed by photos were followed up on by direct field investigations to confirm beaver presence. In particular, we traveled on foot a total of 17.05 km by retracing the same riverine areas 2–3 times (once every three months per area), in the early morning and with wet soil to increase footprint detection, in all rivers where the species was reported to be present.

If beaver signs of presence were detected, we followed [21] for data collection. In particular, we georeferenced all beaver presence signs, including excrements, gnawed trunks, marking sites, lodges, dams, hair, and observed individuals. Gnawed trunks were classified as very old (gnawed over 3 months before our detection), old (gnawed about 1–3 months before our detection), recent (gnawed about 7–30 days before our detection), and very recent (gnawed about 1–7 days before our detection). Where gnawed trunks were detected, two camera traps (©Browning SpecOps) were opportunistically placed near recent gnawing (at heights of ~50–80 cm from the ground, activated for 24 h/day, to take one 30 s video/event [22]) to confirm the presence of this large rodent and to attempt a population assessment. All signs of presence of the Eurasian beaver were included in a global dataset with the coordinates (latitude and longitude) recorded using GPS devices (Garmin ©) with an XY resolution of 10 m, with the number and the typology of presence signs following [22]. The beaver data in the Abruzzo region were collected using camera traps (© Browning Patriot BTC-PATRIOT-FHD) during an otter *Lutra lutra* survey along rivers recently occupied by this species [23].

## 3. Results and Discussion

Between January 2022 and March 2023, we received reliable photos of beaver signs of presence, which were reported to be shot in three areas of Italy:

In Areas 1–2 in Figure 1 in April 2022, two pictures of gnawed trunks were reported for the plain area between Trino Vercellese and Fontanetto Po, on the Po River (Province of Vercelli, Piedmont, Northeast Italy). In the same region, in March 2023, other reports of beaver presence were sent from Migiandone (Ornavasso, Province of Verbano-Cusio-Ossola, Eastern Piedmont, Northwest Italy).

In Area 3 in Figure 1 in December 2022, some doubtful photos of individual swimming beaveres were reported for an area north of Rome (Tevere River).

In Area 4 in Figure 1 in March 2023, gnawed trunks were detected by two authors (Gianmarco Cimorelli and Angelo Casciano) in an area included between the municipalities of Monteroduni-Roccaravindola (province of Isernia, Molise region) and Capriati a Volturno (province of Caserta, Campania), on the Volturno River, in Southern Italy.

**Figure 1:**
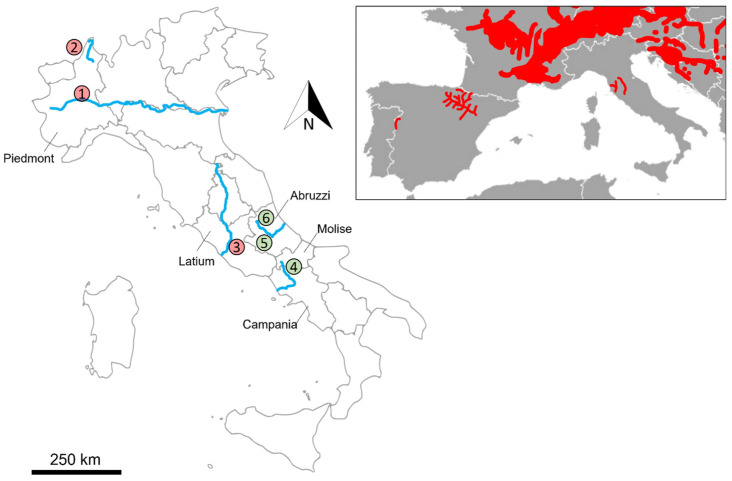
New surveyed areas for beavers: (1) Province of Vercelli, Po River, Piedmont; (2) Migiandone, Toce River, Piedmont; (3) Rome, Tevere River, Latium; (4) Volturno River, Molise-Campania; (5,6) Aterno River, Abruzzi. Red circles refer to unconfirmed records, green ones to confirmed beaver presence. The known distribution of the Eurasian beaver in Italy and neighbouring countries is shown in the inset [1,5,8,9].

The beaver data in the Abruzzo region (Aterno river, Areas 5 and 6 of Figure 1) were collected using camera traps (© Browning Patriot BTC-PATRIOT-FHD) during an otter Lutra lutra survey along rivers recently occupied by this species [23].

**Figure 2:**
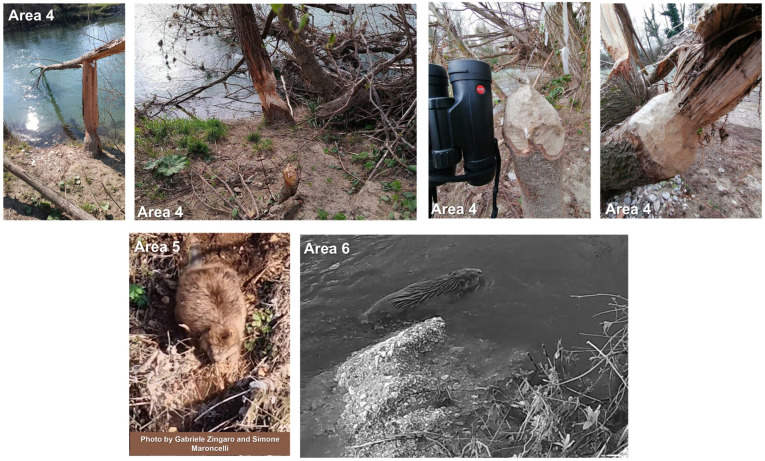
Photos confirming beaver presence in Area 4 (©Gianmarco Cimorelli and Giovanni Capobianco), Area 5 (©Gabriele Zingaro and Simone Maroncelli) and Area 6 (©Romina Fusillo and Manlio Marcelli).

Evidence from Piedmont and the surroundings of Rome was not authenticated, i.e., no sign of beaver presence was detected in any of these locations, at the coordinates reported by the citizen scientist, or in their surroundings. Furthermore, no beaver was recorded by camera traps in these areas. Conversely, the presence of adult and juvenile beavers was immediately confirmed by different sizes of incisive signs and debarking on stems of willows *Salix* spp. and black poplars *Populus nigra* L. along the Volturno River between Campania and Molise (Figure 2). Signs of presence were a mixture of old and recent gnawing, suggesting that beavers may have been present in the study area for at least 2 years. Moreover, camera trapping provided us with further confirmation of the beaver presence, in the framework of the camera-trapping project in the Matese National Park named “Fototrappolaggio Naturalistico nel Matese” (Figure 2).

The beaver population occurring in the Volturno River corresponds to the southernmost population of this species in the world. However, this area is suitable for beaver presence [24], and it also represents the southernmost area where the species occurred in the Middle Ages [6]. In March 2023, three videos, each of them reporting a swimming beaver, were recorded along the Aterno River, in the municipality of Vittorito (province of L’Aquila, Abruzzi). The individual(s) in the videos was/were identified as adults based on body size. However, we did not find yet any biological samples (i.e., excrements, fur) to perform genetic analyses. Moreover, a juvenile individual was reported for the surroundings of L’Aquila, 45 km north of the previous site, still along the Aterno River. Table 1 summarises new occurrences reported in this manuscript.

The Eurasian beaver population is rapidly expanding in Europe following legal protection, habitat recovery, and reintroduction events [1,25,26]. The species was recorded for the first time in Italy in 2018–2020, with individual beavers from the expanding Austrian population reaching two regions of Northeast Italy. In 2021, individuals originating from illegal releases were then recorded in central Italy (Tuscany and Umbria [8,9]). It is unlikely that the beaver occurrences in the Abruzzi (Aterno River) and Campania-Molise regions (Volturno River) derive from releases in central Italy, given that the nearest family group in the central regions is located about 150–200 km northwards, in completely disjointed river basins [24]. Therefore, despite the species being present in the Volturno Valley up to the Middle Ages [6], we suggest that the appearance of the Eurasian beaver in Abruzzi and southern Italy may be due to further unofficial release events (see [24] for a species distribution model in Italy) by wildlife enthusiasts following the example of other European countries [1,5]. No reliable evidence on the number of free-ranging beavers in Southern Italy is available yet, given that we did not find any biological samples (excrement or fur) for genetic analyses [8,9]. Conversely, we collected evidence suggesting that at least one family group is present in our study site, which may represent the first reproductive event for this species in southern regions. Rapid actions for detailed distribution monitoring and assessment of breeding evidence of the species would be necessary to evaluate population establishment (species naturalisation) and its potential range expansion. These actions are fundamental in view of a decision about management steps for these individuals. Removal strategies for unofficially released Eurasian beavers have proven to be both ineffective and expensive (e.g., in Spain [1]). Removal is the first management option to be considered, especially when unauthorized release appears at its early stage and the population is still not established [9]. Nevertheless, the removal option in the Abruzzi and Molise-Campania regions should be carefully evaluated because of the presence of the Eurasian otter in the same rivers where the beavers have been recorded. The Eurasian otter is strictly protected at a national level and listed in Annex II and Annex IV of the Habitats Directive. This species has also been classified as vulnerable (VU) in the recently updated National Red List [11]. Actions to remove beavers can directly disturb otters and lead to accidental deaths [27].

## 4. Conclusions

Beavers are widely recognized as “ecosystem engineers”, and their presence in river habitats can be easily detected by non-specialists, given their conspicuous gnawing behaviour and construction of dams and lodges [28]. Moreover, beavers usually attract human empathy.

A positive attitude towards a species may increase the success of citizen- science initiatives, providing a remarkable increase in its distribution data. Indeed, the intensive media campaign that followed the detection of beavers in central Italy triggered an interest towards this species in Italy, which would have brought an improvement of knowledge on its presence.

In our work, citizen science has played a pivotal role in detecting the southernmost occurrence of beavers in Eurasia. In central and southern Italy, it would be important to concentrate on how to manage the species. Given the distance between the central-southern Italian populations of the Eurasian beaver and the rest of the distribution of the species, it is most likely that the Italian nuclei derive from an unauthorised release. A genetic analysis comparing individuals from Central Italy with those from neighbouring countries (cf. Figure 1) would allow for the determination of the origins of beavers occurring in Italy. Furthermore, a species distribution model complete with a GIS-based analysis of the ecological connectivity and satellite monitoring of some individuals should be recommended to predict the future dispersive dynamics of the species and to rapidly identify isolated, i.e., necessarily translocated, new populations. Removal strategies should carefully consider the pros and cons of such management options in areas where Eurasian otters occur.

In conclusion, our work reveals new Italian hotspots of beaver presence, suggesting the existence of multiple sites of potentially unauthorized releases across the Italian peninsula. Moreover, the discovery in the Volturno River represents the southernmost worldwide suitable habitat for reproductive beavers in Europe. Further monitoring programs should be required to better define the distribution of this large rodent in suitable areas of southern Italian regions.

## Figures and Tables

**Table 1:** New confirmed records of beaver presence in Italy.

Record	River	Region	Minimum Number of Individuals	Latitude	Longitude
1	Aterno, L’Aquila	Abruzzi	One juvenile	42.32° N	13.37° E
2	Aterno, Vittorito	Abruzzi	One adult	42.11° N	13.79° E
3	Volturno, Roccaravindola-Monteroduni	Molise	One juvenile	41.52° N	14.14° E
4	Volturno, Capriati a Volturno	Campania	One family group	41.47° N	14.10° E

## Data Availability

All used data are included within the manuscript.
